# Comparing the Immunogenicity and Protective Effects of Three MERS-CoV Inactivation Methods in Mice

**DOI:** 10.3390/vaccines10111843

**Published:** 2022-10-31

**Authors:** Nayoung Kim, Tae-Young Lee, Hansaem Lee, Jeong-Sun Yang, Kyung-Chang Kim, Joo-Yeon Lee, Hyun-Joo Kim

**Affiliations:** 1 Division of Emerging Virus & Vector Research, Center for Emerging Virus Research, Korea National Institute of Health, Korea Disease Control and Prevention Agency, Cheongju-si 28159, Korea; 2 Division of Infectious Disease Vaccine Research, Center for Vaccine Research, Korea National Institute of Health, Korea Disease Control and Prevention Agency, Cheongju-si 28159, Korea; 3 Center for Emerging Virus Research, Korea National Institute of Health, Korea Disease Control and Prevention Agency, Cheongju-si 28159, Korea

**Keywords:** MERS-CoV, inactivated vaccine, formaldehyde, hydrogen peroxide, BEI, adjuvant, immunogenicity

## Abstract

The Middle East respiratory syndrome (MERS) is a fatal acute viral respiratory disease caused by MERS-coronavirus (MERS-CoV) infection. To date, no vaccine has been approved for MERS-CoV despite continuing outbreaks. Inactivated vaccines are a viable option when developed using the appropriate inactivation methods and adjuvants. In this study, we evaluated the immunogenicity and protective effects of MERS-CoV vaccine candidates inactivated by three different chemical agents. MERS-CoV was effectively inactivated by formaldehyde, hydrogen peroxide, and binary ethylene imine and induced humoral and cellular immunity in mice. Although inflammatory cell infiltration was observed in the lungs four days after the challenge, the immunized hDPP4-transgenic mouse group showed 100% protection against a challenge with MERS-CoV (100 LD_50_). In particular, the immune response was highly stimulated by MERS-CoV inactivated with formaldehyde, and all mice survived a challenge with the minimum dose. In the adjuvant comparison test, the group immunized with inactivated MERS-CoV and AddaVax had a higher immune response than the group immunized with aluminum potassium sulfate (alum). In conclusion, our study indicates that the three methods of MERS-CoV inactivation are highly immunogenic and protective in mice and show strong potential as vaccine candidates when used with an appropriate adjuvant.

## 1. Introduction

The Middle East respiratory syndrome coronavirus (MERS-CoV) was first isolated from a patient in Saudi Arabia in 2012 [1]. MERS-CoV causes severe respiratory disease that leads to pneumonia and renal failure [2]. By the end of February 2022, a total of 2585 laboratory-confirmed cases of Middle East respiratory syndrome (MERS) were reported, including 890 deaths and a mortality rate of 34.4% [3]. In May 2015, a nosocomial outbreak of MERS-CoV occurred in South Korea by an index person from Middle East Asia, resulting in 186 cases at 16 hospitals [4,5].

Nevertheless, MERS outbreaks have continued in Middle East Asia, and there are no licensed vaccines; thus, the development of MERS-CoV vaccine candidates is urgently required worldwide [6]. Ongoing research on vaccine development to prevent MERS infection has been conducted in preclinical studies based on various platforms, such as inactivated, live attenuated, subunit, virus-like particle (VLP), viral vector-recombinant, and DNA [7]. The DNA vaccine showed immunogens of full-length S DNA, and the S1 subunit protein induced strong serum neutralizing activity against MERS-CoV infection in both mice and non-human primate models [8]. Recombinant RBD protein induced a strong and persistent immunological response and reduced viral load in non-human primates [9]. To date, the most preceded MERS-CoV vaccine candidate is based on the chimpanzee adenoviral vector (ChAdOx1) containing the S glycoprotein and is currently in clinical trials [10].

Among the various vaccine platforms, inactivated vaccines have been widely used for the prevention of emerging infectious diseases since the development of the inactivated whole-viral polio vaccine in 1952. Inactivated vaccines are a promising strategy for vaccine development because of their fast development time and low cost [11]. Additionally, this type of vaccine elicits strong humoral and cellular immune responses against viral replication, showing safety in healthy humans [12,13]. To rapidly develop an effective MERS-CoV vaccine, several vaccine studies have been conducted using various inactivation methods, including gamma irradiation and formaldehyde (FA). Despite the hypersensitive lung pathology in both inactivated vaccines, the results suggest that inactivated vaccines are a viable option with appropriate inactivation and adjuvants [14,15].

Chemical viral inactivation is performed using FA, hydrogen peroxide (H_2_O_2_), binary ethylenimine (BEI), or other methods [16]. FA is the most widely used inactivating agent to abolish the infectivity of viruses via protein inactivation, stabilization, or immobilization [17]. The licensed virus vaccines inactivated with FA include poliovirus, hepatitis A virus, and Japanese encephalitis virus (JEV) [18]. H_2_O_2_ is a strong oxidizing agent that can be an effective inactivation method for vaccine production [19]. The inactivated vaccinia virus using H_2_O_2_ induced a stronger virus-specific neutralizing antibody response than the UV-inactivated virus in a BALB/c model [20]. In addition, H_2_O_2_-inactivated West Nile virus (WNV) generated robust adaptive B- and T-cell immune responses in both C57BL/6 and BALB/c mouse models and protected against a challenge with the heterologous virulent North American WNV strain [21]. BEI is an aziridine compound that reacts with viral nucleic acids while maintaining epitope structure and accessibility [22]. BEI-inactivated virus vaccines, including Bursal Disease virus, foot and mouth disease virus, rabies virus, and JEV, were also licensed [23].

In this study, we evaluated the immunogenicity and protective effects of three MERS-CoV inactivation agents—FA, H_2_O_2_, and BEI—in mice. Additionally, we investigated how the immune response differed according to the type of adjuvant used with alum and AddaVax.

## 2. Materials and Methods

### 2.1. Cells and Virus

Vero cells (CCL-81, ATCC, Manassas, VA, USA) were maintained in Dulbecco’s modified Eagle’s medium (DMEM) with 5% fetal bovine serum (FBS), 100 U/mL penicillin, and 100 μg/mL streptomycin at 37 °C in 5% CO_2_. All culture media and supplements were obtained from Thermo Fisher Scientific (Middlesex County, MA, USA). MERS-CoV (MERS-CoV/KOR/KNIH/002_05_2015) was used for plaque assays and virus neutralization tests on Vero cells. All work with live MERS-CoV was performed in Biosafety Level-3 (BSL-3) facilities at the Korea Disease Control and Prevention Agency (KDCA) (Cheongju, Korea).

### 2.2. Virus Inactivation

MERS-CoV supernatants were inactivated with 0.08% FA, 0.3% H_2_O_2_, and 3 mM binary ethylenimine (BEI) for 24 h at 25 °C. The BEI-inactivated virus was neutralized by the addition of 1N sodium thiosulfate. Inactivation of viral infectivity was confirmed using a plaque assay. Viruses inactivated by FA, H_2_O_2_, and BEI were concentrated using polyethylene glycol (PEG; Sigma-Aldrich, St. Louis, MO, USA) and ultracentrifugation at 30,000 rpm for 4 h at 4 °C. The concentrated virus pellet was dissolved in phosphate-buffered saline (PBS) overnight. Inactivated-MERS-CoV was quantified using the PIERCE™ BCA protein assay kit (Thermo Fisher) and stored at −80 °C until use.

### 2.3. Animal Experiments

The 6-week-old female C57BL/6 mice were provided by Daehan Bio Link (Eumseong, Korea) and maintained in an Animal Biosafety Level-2 (ABSL-2) facility at the KDCA. The 6-week-old female hDPP4 (C57BL/6 with human DPP4) transgenic mice were provided by Macrogen (Seoul, Korea) and were maintained in an ABSL-3 facility at the KDCA. All experimental procedures were approved by the Institutional Animal Care and Use Committee (KDCA-IACUC-20-041) and conducted in accordance with the KDCA guidelines. All animal experimental groups for comparing the immunogenicity and protective effects of three inactivated MERS-CoV are presented in Table S1.

#### 2.3.1. Immune Responses in C57BL/6 Mice (Experiment 1)

To investigate immunogenicity, C57BL/6 mice were randomly divided into three groups (*n* = 5 per group) and intramuscularly immunized with 10 μg of inactivated MERS-CoV and aluminum potassium sulfate (alum) three times at two-week intervals. The control group (*n* = 5) was immunized with alum alone. Sera were obtained by orbital blood sampling before each immunization. Two weeks after the last immunization, all mice were euthanized, and splenocytes were collected for Enzyme-linked immunospot (ELISPOT).

#### 2.3.2. Protection Effect in hDPP4 Transgenic Mice (Experiment 2)

To examine the protective effect against viral infection, three groups (*n* = 25 per group) of hDPP4-transgenic mice were immunized, as described in Section 2.3.1. The control group (*n* = 25) was immunized with only alum alone. Sera were obtained by orbital blood sampling before each immunization. After two weeks, mice were intranasally infected with 100 LD_50_ (1 × 10^5^/30 μL) MERS-CoV. For intranasal infection, mice were anesthetized by intraperitoneal (IP) with a mix of 30 mg/kg Zoletil^®^ (Virbac, St. Louis, MI, USA) and 10 mg/kg Rompun^®^ (Bayer, Berkeley, CA, USA). Challenged mice were euthanized on days 4 and 8 post-infection (10/group/day) for the analysis of lung pathology and virus titer. The body weight and survival rates of the remaining mice (*n* = 5) were measured, and the mice were euthanized two weeks after viral infection. Sera were obtained by orbital blood sampling before each immunization.

#### 2.3.3. Comparison of Dose and Number in hDPP4 Transgenic Mice (Experiment 3)

To optimize the dose and number of immunizations for the best immunogenicity and safety, hDPP4 mice were divided into nine groups (*n* = 5 per group). The G1 to G3 were immunized twice with FA-inactivated MERS-CoV at doses of 1, 5, or 10 μg, respectively, in the presence of alum at two-week intervals. H_2_O_2_ (G4, G5, and G6), BEI (G7, G8, and G9)-inactivated MERS-CoV were also immunized using the same methods. The control group (G10, *n* = 5) was immunized with alum alone. Sera were obtained by orbital blood sampling before each immunization. After the two weeks, all mice were challenged with live MERS-CoV at 100 LD_50_ as described in Section 2.3.2. Their body weight and survival rate were measured for further two weeks and finally all mice were euthanized.

Additionally, to determine the minimum dose, hDPP4 mice were divided into three groups (*n* = 5 per group). Mice were intramuscularly immunized once with 1 μg of three inactivated MERS-CoV with alum. After two weeks, all mice were challenged and euthanized using the same methods as described above.

#### 2.3.4. Comparison of Adjuvant in hDPP4 Transgenic Mice (Experiment 4)

The hDPP4 mice were divided into nine groups (*n* = 5 per group). Each group was immunized twice with FA-inactivated MERS-CoV at 1, 5, or 10 μg dosage, respectively, without adjuvant (G1, G2, and G3) or with alum (G4, G5, and G6) or AddaVax^®^ (Invitrogen, San Diego, CA, USA) (G7, G8, G9). The control group (G10, *n* = 5) was immunized with only alum. After that, all mice were challenged and euthanized, as described in Section 2.3.3.

### 2.4. Histology

After euthanasia, the lungs were collected, fixed overnight in zinc formalin, and embedded in paraffin. Lung sections were stained with hematoxylin and eosin, and infiltrating immune cells (mononuclear cells and eosinophils) were assessed using light microscopy. For pathological diagnosis, reference was made to the International Harmonization of Nomenclature and Diagnostic Criteria (INHAND) [24].

### 2.5. Enzyme-Linked Immunosorbent Assay (ELISA)

The 96-well Maxisorp plates (Thermo Fisher Scientific) were coated with 100 ng of MERS-CoV nucleocapsid protein (NP; Sinobio, Beijing, China) and spike protein (S1 subunit; Sinobio) in PBS overnight at 4 °C. Plates were blocked with 100 μL Tris-buffered saline with 0.01% tween-20 (TBS-T) and 5% skim milk for 1 h at 37 °C. After washing with TBS-T, plates were incubated with serum (100 μL/well) diluted 3-fold at 1:1000 to 1:243,000 prepared in TBS-T with 3% skim milk for 1 h at 37 °C. Plates were washed and incubated with anti-mouse IgG/IgG2c/IgG1-HRP (Sigma-Aldrich) at 1:5000 dilutions in TBS-T with 3% skim milk for 1 h at 37 °C. Plates were developed using a tetramethyl benzidine (TMB; Thermo Fisher) substrate and stopped with a stop solution (Thermo Fisher). Absorbance was measured at 450 nm using a microplate reader (VICTOR Nivo™; PerkinElmer, Waltham, MA, USA).

### 2.6. Enzyme-Linked Immunospot (ELISPOT)

Mouse splenocytes were isolated from spleen tissues, as previously reported [25]. The mouse IFN-γ ELISPOT kit (BD Biosciences, San Jose, CA, USA) was used to detect IFN-γ-secreting T-cells. Briefly, mouse splenocytes were seeded at 1 × 10^6^ cells/well in ELISPOT plates and stimulated with 10 µg/well inactivated MERS-CoV for 24 h at 37 °C. Spot numbers were counted using ImmunoSpot^®^ (Cellular Technology Limited, Cleveland, OH, USA) and analyzed using GraphPad Prism version 7 (GraphPad Software, San Diego, CA, USA).

### 2.7. Virus Titration (Plaque Assay)

To analyze virus titers, lung and brain tissues were homogenized with beads and Precellys (Bertin, le-Bretonneux, France). Homogenized tissues were centrifuged, and supernatants were collected. Vero cells were seeded at a density of 5 × 10^5^ cells/well in 6-well plates. The cells were infected with 10-fold serially diluted tissue samples and incubated for 1 h at 37 °C. After incubation, cells were covered with low-melting agarose (Lonza, Basel, Switzerland) overlay, including 1 × MEM supplemented with 2% FBS. After subsequent incubation for four days, cells were stained with 0.2% crystal violet solution (Sigma-Aldrich), and plaques were counted.

### 2.8. Virus Neutralization Test (VNT)

Mouse sera were inactivated at 56 °C for 30 min and serially diluted from 1:20 to 1:40,960 in DMEM. Diluted sera were incubated with equal volumes of 100 tissue culture infectious dose 50% (TCID_50_) of MERS-CoV for 1 h at 37 °C. After incubation, 1 × 10^4^ Vero cells in 50 μL of DMEM were added to the sera and virus mixture. Plates were incubated for four days at 37 °C, and the presence of cytopathic effects was observed under a light microscope. All sera were tested in duplicate.

### 2.9. Statistical Analysis

All data are expressed as means ± standard error of the mean (SEM). Statistical significance was determined using the Student’s *t*-test and one-way ANOVA multiple test followed by Dunnett’s post-test (Prism; GraphPad Software). Statistical significance was set at *p* < 0.05.

## 3. Results

### 3.1. Immunogenicity of Inactivated-MERS-CoV in C57BL/6 Mice

**Figure 1:**
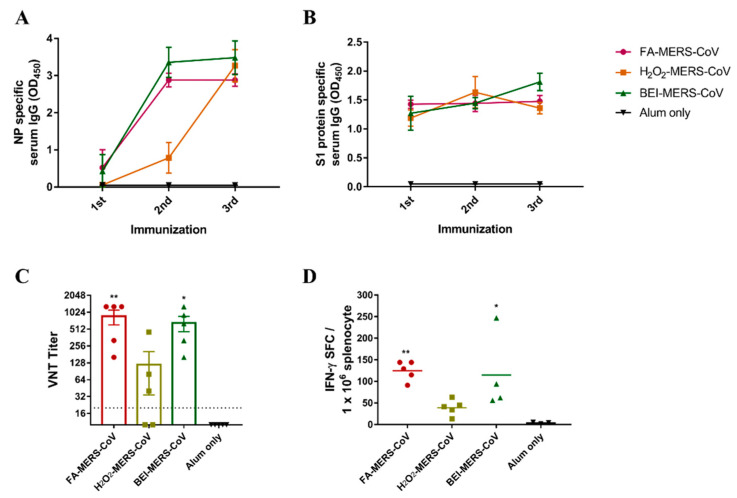
Inactivated MERS-CoV induced humoral/cellular immune responses in C57BL/6 mice. MERS-CoV nucleocapsid protein (**A**) and S1 protein-specific serum IgG (**B**) levels were measured by ELISA. Serum was collected after the first, second, and third immunization at two-week intervals. (**C**) MERS-CoV specific neutralizing antibodies (nAbs) were determined by virus-neutralizing titers (VNT). Serum was collected two weeks after the third immunization. (**D**) IFN-γ secreting T-cell were quantified by ELISPOT. The splenocytes from last immunized mice were stimulated with inactivated-MERS-CoV. Data are represented as the mean ± S.E.M. * *p* < 0.05, ** *p* < 0.01 compared with alum only.

The minimum concentration that can inactivate MERS-CoV was confirmed in a previous study (not reported). MERS-CoV was successfully inactivated using the three chemicals. To evaluate whether MERS-CoV inactivated by the three methods induced an immune response, inactivated cells with alum were immunized intramuscularly three times at two-week intervals in C57BL/6 mice. As shown in Figure 1A,B, IgG specific for MERS-CoV NP and S1 protein was induced in all immunized mice by ELISA. After the second booster of FA- and BEI-inactivated MERS-CoV, antibody levels of the NP protein significantly increased by approximately 5.5 and 8 times, respectively. In contrast, the antibody levels in the H_2_O_2_ group increased after the third booster (Figure 1A). The S1 antibody level showed little difference between the second and third boosters in the FA group but showed a gradual increase in the BEI group (Figure 1B). Furthermore, similar to the ELISA results, neutralizing antibodies in the FA and BEI groups were higher than those in the H_2_O_2_ group (Figure 1C).

To analyze the cellular immunity induced by inactivated MERS-CoV, IFN-γ-secreting T-cells were analyzed using ELISPOT (Figure 1D). This showed that higher cellular immunity was induced by FA- and BEI-inactivated MERS-CoV compared to H_2_O_2_.

### 3.2. Protection Effect of Inactivated MERS-CoV in hDPP4 Transgenic Mice

**Figure 2:**
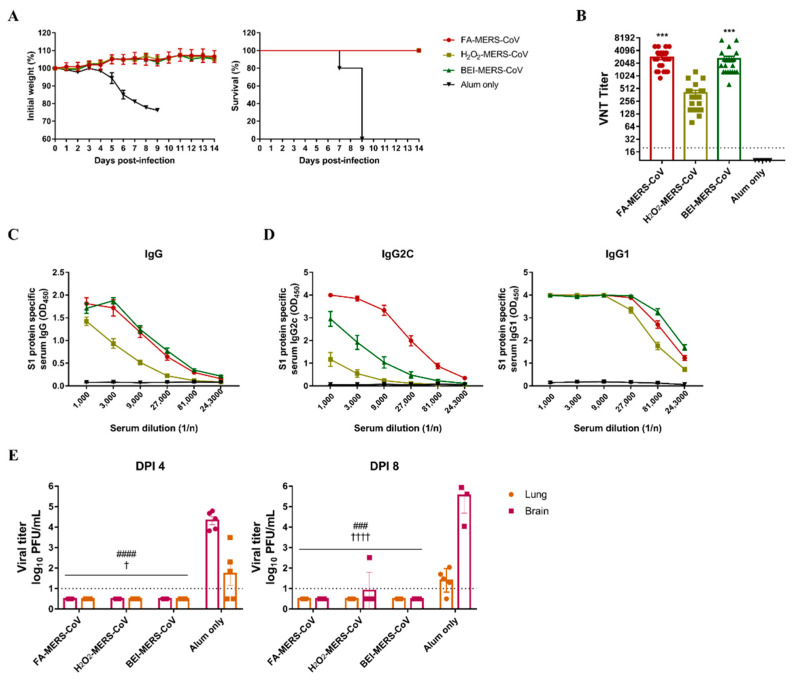
The three inactivated MERS-CoV induced a protective immune response against MERS-CoV infection in the hDPP4-transgenic mouse model. (**A**) Changes in body weight and survival rate were monitored for two weeks after MERS-CoV infection. The survival symbols of H_2_O_2_-MERS-CoV and BEI-MERS-CoV overlapped with FA-MERS-CoV. (**B**) MERS-CoV specific neutralizing antibodies (nAbs) and (**C**,**D**) S1 protein-specific serum IgG/IgG2c/IgG1 levels were measured for two weeks after the third immunization. (**E**) MERS-CoV titers in lung and brain tissues were observed by plaque assay on dpi 4 and 8 after the MERS virus challenge. Data are represented as the mean ± S.E.M. *** *p* < 0.001 compared with alum only. ### *p* < 0.001, #### *p* < 0.0001 compared with alum only lung. † *p* < 0.05, †††† *p* < 0.00001 compared with alum only brain.

As the immune response was induced in C57BL/6 mice by inactivated MERS-CoV, the protective effect against infection was evaluated using hDPP4 transgenic mice. All immunized groups survived and lost weight (Figure 2A). Before the MERS-CoV challenge, the neutralizing antibody and S1 specific IgG antibody titers were evaluated and showed higher levels in the FA and BEI groups than in the H_2_O_2_ group (Figure 2B,C). To determine the immune pathway, we measured antibody titers of IgG2c and IgG1a. As shown in Figure 2D, the FA group had the highest level of IgG2c antibody involved in the Th1 immune response, whereas IgG1, which is involved in the Th-2 reaction, was not significantly different among the three methods.

Mice were euthanized four and eight days after the MERS-CoV challenge, and viral titers in the lungs and brains were measured. No virus was detected in the tissues of the immunized groups except for one mouse in the H_2_O_2_ group on day eight (Figure 2E). These results demonstrate that the three inactivated MERS-CoV strains can induce a potent immune response to protect against viral infections.

**Figure 3:**
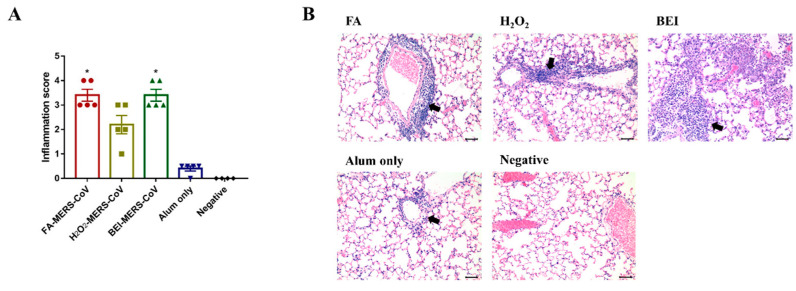
Histopathological changes in the lungs of immunized groups. (**A**) Inflammation score of lung pathology. (**B**) Representative results of hematoxylin-eosin (H&E) staining (×200) in the lungs of immunized mice, alum only (positive) or negative. Note the heavy infiltration of inflammatory cells around the blood vessels (arrows). Bars = 50 μm. Data are represented as the mean ± S.E.M. * *p* < 0.05 compared with alum only.

Histopathological changes in the lungs were noted in all immunized groups four days after the MERS-CoV challenge. The lungs of mice immunized with inactivated MERS-CoV showed inflammatory cell foci with monocytes and eosinophils. On a severity scale of 0 to 4 (none, minimal, mild, moderate, severe), the inflammation score was similar in the FA and BEI groups (3.4 ± 0.55), and the lowest score was observed in the H_2_O_2_ group (2.2 ± 0.84) (Figure 3A). Infiltration was observed in the perivascular area adjacent to the bronchioles and lung parenchyma (Figure 3B).

### 3.3. Comparing Dose and Number of Immunizations

**Figure 4:**
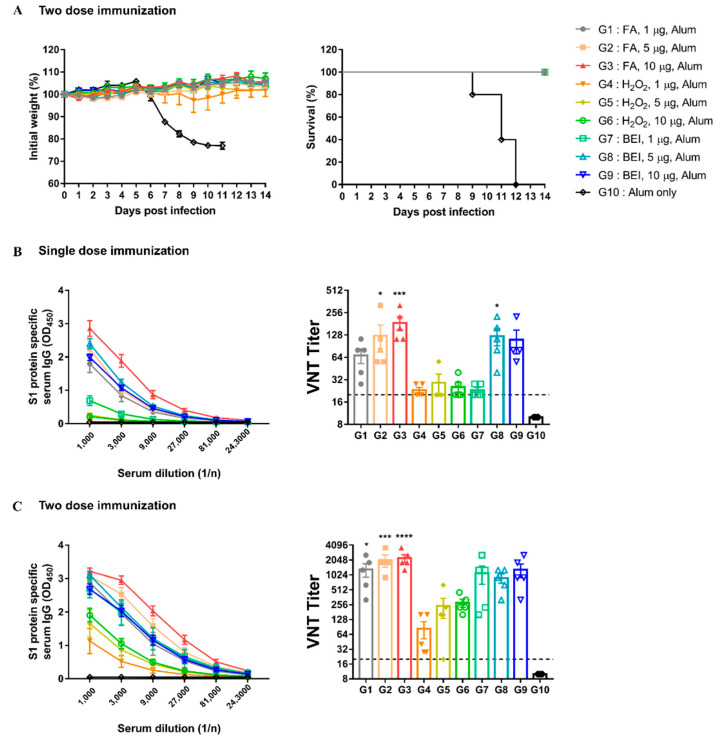
Inactivated-MERS-CoV by formaldehyde has higher protective effect for MERS-CoV than H_2_O_2_ and BEI. (**A**) Changes in body weight and survival rate for 2 weeks when infected with the virus after two dose immunization. The survival symbols of G2 to G9 overlapped with G1. (**B**) MERS-CoV S1 protein-specific serum IgG levels and nAbs were measured by ELISA and VNT. Serum was collected 2-weeks after single dose immunization. (**C**) MERS-CoV S1protein-specific serum IgG levels and nAbs after two dose immunization. Data are represented as the mean ± S.E.M. * *p* < 0.05, *** *p* < 0.001, **** *p* < 0.0001 compared with G10 (Alum only).

To optimize the dose and number of immunizations for the best immunogenicity and safety, hDPP4 mice were immunized with different doses of inactivated MERS-CoV once or twice at two-week intervals. After the second immunization, all immunized groups (G1 to G9) survived the challenge, except for the G10 mock-treated control (Figure 4A). As shown in Figure 4B,C, the antibody response increased as the dose and immunization number increased. In addition, the S1 protein-specific IgG and neutralizing antibody titers of the FA (G1 to G3) and BEI groups (G7 to G9) were higher than those in the H_2_O_2_ groups (G4 to G6) at the same dose (Figure 4B,C). Particularly, in the FA group, the neutralizing antibody titer was more than four times higher than that of other groups when 1 μg was immunized.

To determine the minimum dose at which a protective immune response was induced, we conducted an additional experiment. Therefore, only 1 μg of the FA group showed a 100% survival rate without weight loss (Figure S1). However, in the other groups, survival rates were less than 20%, and a loss of body weight was also observed. These results suggest that 1 μg of FA-inactivated MERS-CoV was sufficient to induce potent immune responses in immunized mice.

Altogether, these data show that the second immunization with inactivated MERS-CoV effectively induced an immune response, especially in the FA group. Moreover, it suggests that FA-inactivated MERS-CoV can be safely used because it shows protective ability with only one immunization at a minimum dose.

### 3.4. Comparison of Adjuvant Test

To enhance the immunogenicity of inactivated MERS-CoV, AddaVax and alum were used as adjuvants, and their ability to induce immune responses was compared in the hDPP4 model. FA-inactivated MERS-CoV was selected for an adjuvant comparison because a good immune response was induced in the previous results (Section 3.2 and Section 3.3). Experiments were performed using various doses of immunization, boosting, and adjuvants (Table S1).

**Figure 5:**
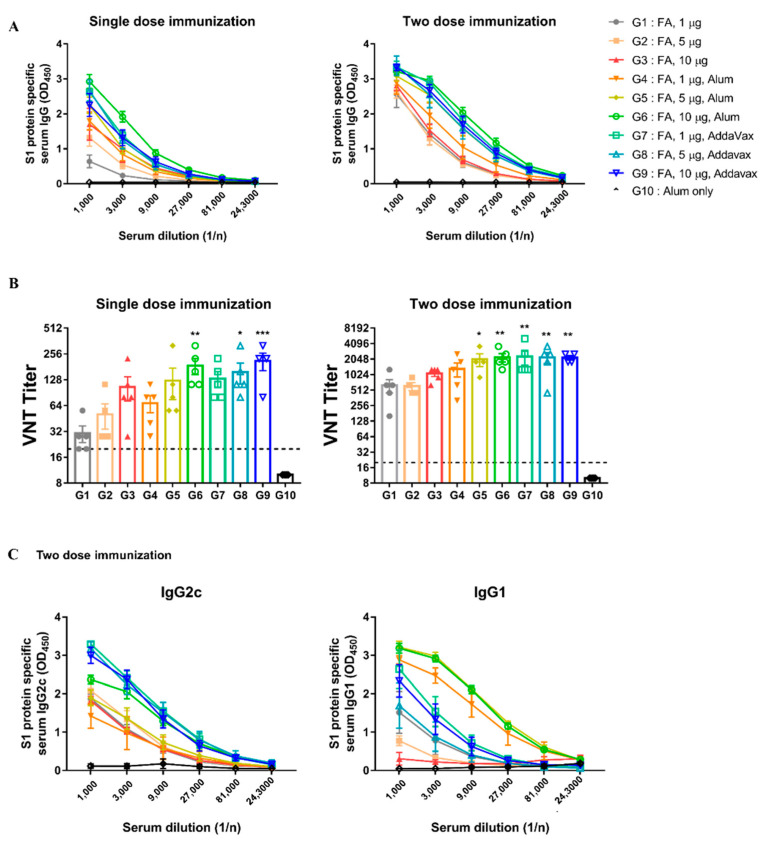
Immunization with inactivated-MERS-CoV with AddaVax has more effective protection against MERS-CoV. (**A**) MERS-CoV S1 protein-specific serum IgG levels were measured by ELISA. Serum was collected 2 weeks after immunization. (**B**) MERS-CoV specific neutralizing antibodies (nAbs) were determined by VNT. Serum was collected 2 weeks after immunization. (**C**) S1 protein-specific serum IgG2c/IgG1 levels were measured for two weeks later after two doses of immunization. Data are represented as the mean ± S.E.M. * *p* < 0.05, ** *p* < 0.01, *** *p* < 0.001 compared with G10 (Alum only).

All immunized groups, with or without adjuvants, survived and showed no weight loss (Figure S2). In the adjuvant groups (G4 to G9), S1 specific IgG and neutralizing antibody titers were higher than those in the groups without adjuvants (G1 to G3) at the same dose (Figure 5A,B). As shown in Figure 5B, the AddaVax groups exhibited higher neutralizing antibody levels than the alum groups at the same dose, regardless of the number of immunizations. To evaluate the effect of the adjuvant on Th1 and Th2 immune responses, we measured antibody titers of IgG2c and IgG1a. We found that the AddaVax groups had higher IgG2c levels involved in the Th1 than the alum groups (Figure 5C). Overall, these results suggest that FA-inactivated MERS-CoV was immunogenic by itself; however, the addition of adjuvants improved its immunogenicity depending on the type of adjuvant used. In particular, AddaVax appeared to be a more potent adjuvant than alum in this study.

The most effective protection was obtained when FA-inactivated MERS-CoV was immunized twice with the AddaVax adjuvant.

## 4. Discussion

In this study, we evaluated the immune response and protective effects of MERS-CoV inactivated by three chemical agents. Although various vaccine platforms, including whole viruses, DNA, subunits, and recombinant viruses, are currently being developed, inactivated vaccines are still being studied as potential vaccine candidates because of several advantages—relatively low production cost, good safety profile, and lack of need for laborious genetic manipulation. Deng et al. showed that FA-inactivated MERS-CoV with alum and CpG ODN protected hDPP4 mice from MERS-CoV infection [15]. Wirblich et al. also reported that inactivated rabies virus containing the MERS-CoV spike protein showed safe and protective effects against MERS-CoV infection in hDPP4 transgenic mice [26]. As expected, our data demonstrate the potential of inactivated MERS-CoV as a vaccine candidate.

MERS-CoV was completely inactivated by the three chemicals and induced humoral and cellular immune responses in C57BL/6 mice. We examined whether Th1 or Th2 immune pathways were induced by inactivated MERS-CoV in hDDP4 transgenic mice. It has been reported that Th1-dependent IFN-γ was associated with the production of IgG2a, and the Th2 cytokine IL-4 stimulated the expression of IgG1 in mice [27]. However, since C57BL/6 mice express the IgG2c isotype instead of IgG2a [28], we measured IgG2c titers in hDPP4 mice. The FA group showed the highest level of IgG2c antibody, whereas the IgG1 antibody level was not significantly different between the three methods. A balanced response ratio of Th1 and Th2, or the Th1-based immune response, is known to be more effective against viral infection. These results suggest that MERS-CoV inactivated by FA is more effective against viral infection than other inactivated methods by inducing a balanced Th1/Th2 immune response.

FA is an alkylating and crosslinking agent that causes gene and protein modifications and is known to damage antigenic epitopes, leading to reduced immunogenicity. H_2_O_2_ is an oxidizing agent that causes genomic damage by hydroxyl radical attack, whereas BEI is an alkylating agent that does not influence the virus structure at low concentrations [16]. When developing an inactivated vaccine, it is important to maintain the viral structure; thus, it is better to choose a method that acts on the viral genome, such as H_2_O_2_ or BEI. However, as mentioned above, several studies using FA-inactivated MERS-CoV have confirmed that immune responses are well induced. Amanna et al. reported that H_2_O_2_-inactivated vaccines caused minimal damage to viral epitopes and induced neutralizing antibodies in mice compared to formalin-inactivated vaccines [20]. However, in this study, H_2_O_2_-inactivated MERS-CoV showed a weaker immune response in hDPP4 mice. Our study differs from that of Amanna et al. in several respects. They used FA and H_2_O_2_ at concentrations of 1%, while we used minimum virus inactivation concentrations of 0.08% and 0.3%, respectively. We assumed that this difference might be caused by greater damage to the epitopes of MERS-CoV antigen by the relatively higher concentration of H_2_O_2_ compared to FA and BEI in this study.

Previously, it was reported that inactivated severe acute respiratory syndrome (SARS)-CoV and MERS-CoV resulted in hypersensitive lung immunopathological reactions with eosinophil infiltration after wild-type virus challenge in mice [14,29]. Li et al. also confirmed that more eosinophils were observed in the lungs of mice immunized with inactivated MERS-CoV compared with a parainfluenza virus (PIV) 5-based vaccine expressing the MERS-CoV envelope spike protein (PIV5/MERS-S) [30]. Eosinophil infiltration induced by the Th2 immune response may be considered a risk indicator of a hypersensitive response when applied to humans. In our histological results, inflammatory cell infiltration was observed in the lungs of the inactivated MERS-CoV groups, as previously reported. However, the H_2_O_2_ group showed a lower inflammation score than the other groups, suggesting the inflammatory response differs according to the inactivation method. In the immunized group, the virus was not detected and showed sufficient protective immune responses, whereas, in the alum-only group (positive control group), the virus was detected at a high titer (10^4^ PFU/mL) and almost no immune response. These results suggest that inactivated MERS-CoV has potential as a vaccine candidate, although lung hypersensitivity reactions were observed.

Additionally, we speculated that there might be more hypersensitive lung pathology with three high-dose alum immunizations. Alum is the most commonly used adjuvant for human vaccination; however, it induces a strong Th2 response via the depot effect and activation of antigen-presenting cells [31]. Deng et al. used a combination of two adjuvants to induce both Th1 and Th2 immune responses [15]. They did not observe lung pathology with formalin-inactivated MERS-CoV adjuvant with alum and oligodeoxynucleotides containing unmethylated CpG (CpG-OND) motifs. Thus, different adjuvants, inactivation methods, and animal models may induce different immune responses to inactivated MERS-CoV. Craig et al. also stated that there are various options for an appropriate inactivation method and adjuvant for the development of inactivated virus vaccines [32]. Therefore, we used AddaVax and alum to determine the effect of the adjuvant on inactivated MERS-CoV. AddaVax is a squalene-based oil-in-water nanoemulsion based on the MF59 formulation. Unlike alum, which induces a strong Th2 response, squalene oil-in-water emulsions, such as MF59, are known to enhance the immune response without being biased towards Th1 or Th2 [33]. Thus, our results showed a higher Th1 immune response, which is important for protection of viral infection, in AddaVax groups than that in alum groups. In a previous study, the antibody and neutralizing antibody titers increased, while the virus number decreased in lung cells after MERS virus infection with MF59 as an adjuvant for the MERS virus spike protein RBD recombinant antigen [34]. Similarly, in our study, it was confirmed that the immune response induced by FA-inactivated MERS-CoV with AddaVax was stronger than that of alum. Our results suggest that AddaVax is a more potent adjuvant than alum for FA-inactivated MERS-CoV.

A limitation of this study was that we only compared FA-inactivated MERS-CoV with two types of adjuvants and did not evaluate lung pathology. Although the difference in lung pathology caused by the adjuvants alum and MF59 was not confirmed [14], it has been reported that eosinophilic infiltration was reduced in SARS-CoV when the Toll-like receptor adjuvant was used [35]. Recently, several adjuvants have been reported, such as single-stranded RNA acting as an agonist for TLR 7/8 and nanoparticles designed with aminated β-glucan and CpG-OND [36,37]. These studies suggest that adjuvants can be used in various ways to increase the safety and immunity of inactivated viruses. We expect that inactivated MERS-CoV with suitable adjuvants can reduce inflammatory conditions in the lung, and further studies might be necessary to fully understand their correlation.

## 5. Conclusions

To our knowledge, this is the first study to compare the immune responses of three different MERS-CoV inactivation chemicals. Taken together, our results demonstrated that inactivation of MERS-CoV with FA, H_2_O_2_, or BEI induced effective immune responses, particularly FA, and could be used as vaccine candidates despite lung pathology. Moreover, the combination of FA-inactivated MERS-CoV and the AddaVax adjuvant enhanced the protective effect against MERS virus infection.

## Data Availability

Not applicable.

## Supplementary Materials

The following supporting information can be downloaded at: https://www.mdpi.com/article/10.3390/vaccines10111843/s1, Table S1: Animal experimental groups for comparing the immunogenicity and protective effects of three inactivated MERS-CoV; Figure S1: Comparison of body weight and survival rate in the minimum dose test; Figure S2: Comparison of body weight and survival rate in adjuvant test.
