# Supplementary material for: Comparing the Immunogenicity and Protective Effects of Three MERS-CoV Inactivation Methods in Mice
Source: Vaccines (Basel). 2022 Oct 31;10(11):1843. doi: 10.3390/vaccines10111843 (PMC9693512; doi:10.3390/vaccines10111843)
Supplement: Supplementary file 1 [file vaccines-10-01843-s001.zip › vaccines-1940301-supplementary.pdf]

**Table S1. Animal experimental groups for comparing the immunogenicity and protective effects of three inactivated MERS-CoV**

| Group | Experiment 1<br>Immune response<br>(n = 5) | Experiment 2<br>Protection effect<br>(n = 25) | Experiment 3.1<br>Comparison of dose and number<br>(n = 5) | Experiment 3.2<br>Comparison of minimum dose<br>(n = 5) | Experiment 5<br>Comparison of adjuvant<br>(n = 5) |
|---|---|---|---|---|---|
| 1 | FA, 10 μg, alum | FA, 10 μg, alum | FA, 1 μg, alum | FA, 1 μg, alum | FA, 1 μg |
| 2 | $H_2O_2$, 10 μg, alum | $H_2O_2$, 10 μg, alum | FA, 5 μg, alum | $H_2O_2$, 1 μg, alum | FA, 5 μg |
| 3 | BEI, 10 μg, alum | BEI, 10 μg, alum | FA, 10 μg, alum | BEI, 1 μg, alum | FA, 10 μg |
| 4 | alum | alum | $H_2O_2$, 1 μg, alum | alum | FA, 1 μg, alum |
| 5 | | | $H_2O_2$, 5 μg, alum | | FA, 5 μg, alum |
| 6 | | | $H_2O_2$, 10 μg, alum | | FA, 10 μg, alum |
| 7 | | | BEI, 1 μg, alum | | FA, 1 μg, AddaVax |
| 8 | | | BEI, 5 μg, alum | | FA, 5 μg, AddaVax |
| 9 | | | BEI, 10 μg, alum | | FA, 10 μg, AddaVax |
| 10 | | | alum | | alum |

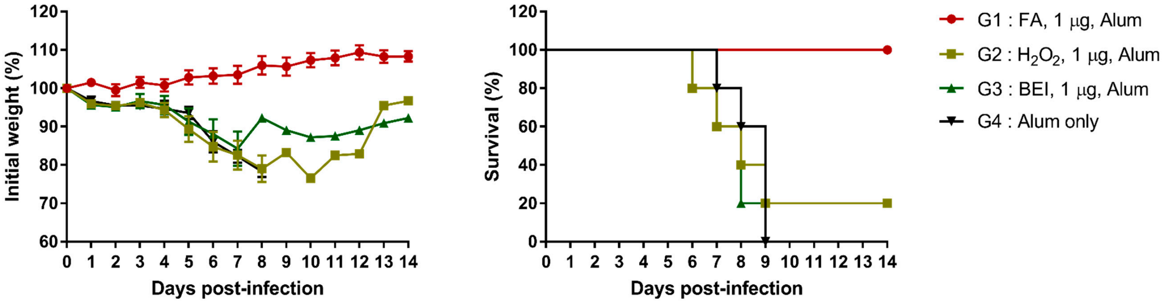


**Figure S1. Comparison of body weight and survival rate in the minimum dose test.** Changes in body weight and survival rate for 2 weeks when infected with the virus after single dose immunization.

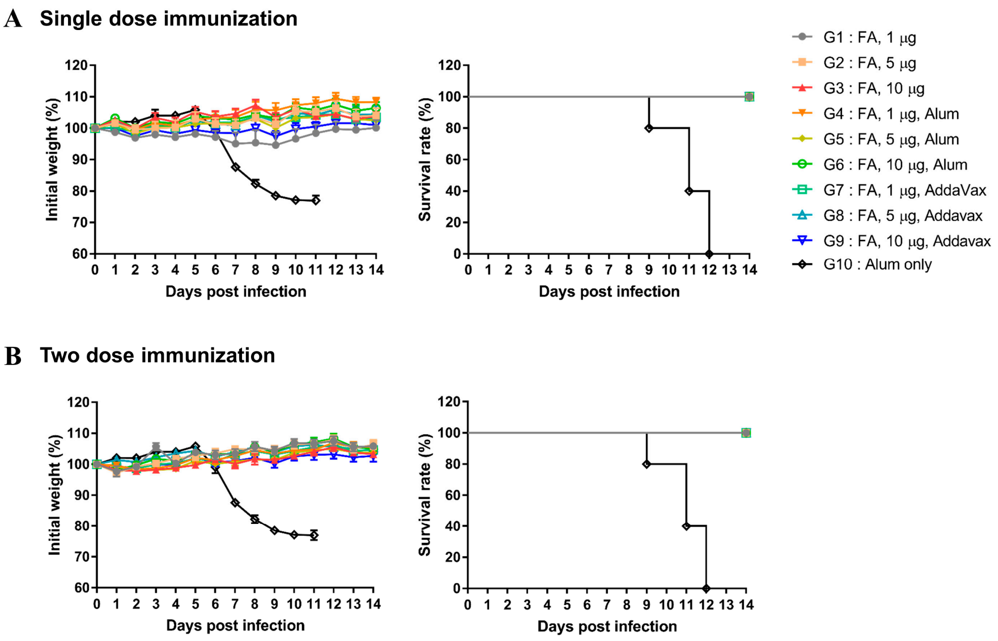


**Figure S2. Comparison of body weight and survival rate in adjuvant test.** (A) Changes in body weight and survival rate for 2 weeks when infected with the virus after single dose immunization. (B) Changes in body weight and survival rate for 2 weeks when infected with the virus after two dose immunization. The survival symbols of G2 to G9 overlap with G1.
